# The medicinal chemistry of *Urtica dioica* L.: from preliminary evidence to clinical studies supporting its neuroprotective activity

**DOI:** 10.1007/s13659-023-00380-5

**Published:** 2023-05-12

**Authors:** Prabhakar Semwal, Abdur Rauf, Ahmed Olatunde, Pooja Singh, Mohamed Y. Zaky, Md. Mozahidul Islam, Anees Ahmed Khalil, Abdullah S. M. Aljohani, Waleed Al Abdulmonem, Giovanni Ribaudo

**Affiliations:** 1 Department of Biotechnology, Graphic Era Deemed to be University, 566/6 Bell Road, Clement Town, Dehra Dun, India; 2 Department of Chemistry, University of Swabi, Swabi, Pakistan; 3 Department of Medical Biochemistry, Abubakar Tafawa Balewa University, Bauchi, Nigeria; 4 Molecular Physiology Division, Faculty of Science, Beni-Suef University, Beni-Suef, Egypt; 5 Oncology Division, Department of Biomedical and Clinical Science, Faculty of Medicine, Linköping University, Linköping, Sweden; 6 Department of Environmental Management, SESM, Independent University, Bangladesh, Bashundhara R/A, Dhaka, Bangladesh; 7 University Institute of Diet and Nutritional Sciences, Faculty of Allied Health Sciences, The University of Lahore, Lahore, Pakistan; 8 Department of Veterinary Medicine, College of Agriculture and Veterinary Medicine, Qassim University, Buraydah, Saudi Arabia; 9 Department of Pathology, College of Medicine, Qassim University, Buraydah, Saudi Arabia; 10 Department of Molecular and Translational Medicine, University of Brescia, Brescia, Italy

**Keywords:** *Urtica dioica*, Neuroprotection, Natural compounds, Flavonoids, Medicinal chemistry

## Abstract

*Urtica dioica* is a perennial herb from the family of Urticaceae that is commonly known as stinging nettle. This plant is widespread in Europe, Africa, America, and a part of Asia, as it adapts to different environments and climatic conditions. The leaves, stalk, and bark of *U. dioica* found applications in the field of nutrition, cosmetics, textile, pest control and pharmacology. In this connection, bioactive chemical constituents such as flavonoids, phenolic acids, amino acids, carotenoids, and fatty acids have been isolated from the plant. With this review, we aim at providing an updated and comprehensive overview of the contributions in literature reporting computational, in vitro, pre-clinical and clinical data supporting the therapeutic applications of *U. dioica*. Experimental evidence shows that *U. dioica* constituents and extracts can provide neuroprotective effects by acting through a combination of different molecular mechanisms, that are discussed in the review. These findings could lay the basis for the identification and design of more effective tools against neurodegenerative diseases.

**Graphical Abstract:**

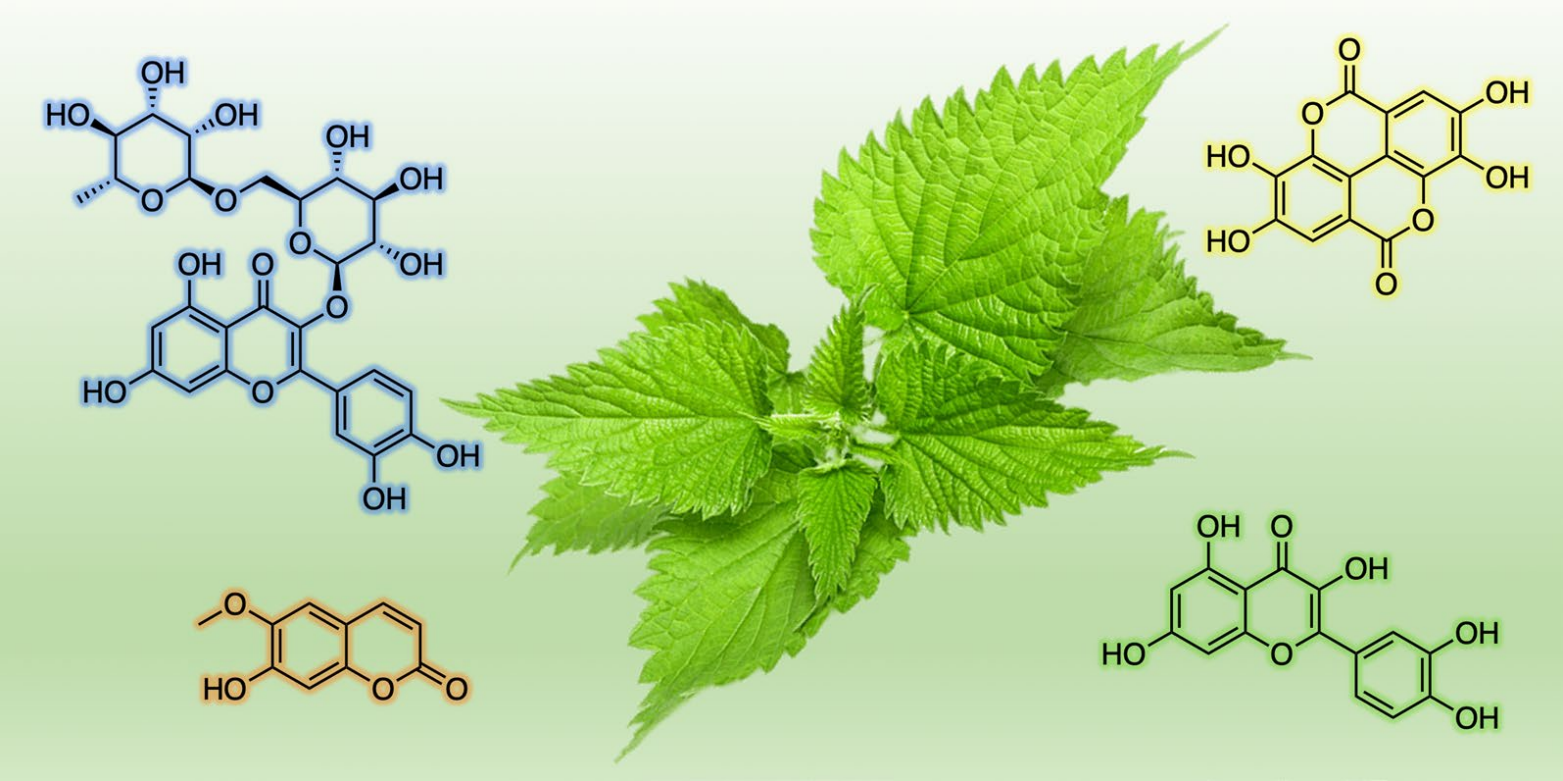

## Introduction

Stinging nettle (*Urtica dioica* L.) is a common wild vegetable that has been known for centuries, and this perennial herbaceous plant from the Urticaceae family is widespread almost worldwide, with a higher presence in Europe, North America, North Africa, and some regions of Asia [1, 2].

Throughout centuries and continents, *U. dioica* was employed in several fields. Besides its well-known role in the context of food and nutrition, it found application, in the form of a slurry, as a fertilizer for several plantations [3, 4]. This plant and its extracts can also be commonly found in cosmetic preparations, and bast fibers can be used for the production of textiles [5, 6]. Eventually, *U. dioica* is a source of chlorophyll, a food coloring ingredient (E140) used in the food and pharmaceutical industry [7, 8].

On the other hand, *U. dioica* also represents a pharmacologically relevant source of bioactive compounds. In ethnopharmacology, it is considered a plant with therapeutic beneficial properties, capable of both preventing and treating illnesses. It is traditionally used for the cure of hypertension, gastrointestinal and hepatic disorders and diabetes [9], but a detailed study of the pharmacological profile and of the chemical constituents was undertaken only more recently [10–13]. More specifically, terpenoids, sphingolipids, steroids, lignans, flavonoids and other alkaloids represent the main bioactive constituents identified in *U. dioica* [5, 14–16].

According to currently available reports, *U. dioica* has been shown to possess antioxidant, hypotensive, anti-inflammatory, anti-diabetic, analgesic, antioxidant and antiproliferative properties [14, 17–19]. In this connection, *U. dioica* was also studied alone and in combination as a remedy against benign prostatic hyperplasia thanks to its antioxidant and anti-inflammatory properties [20–22]. Moreover, its role in preventing the development of cardiovascular disease has been explored [11, 23–26]. Additionally, *U. dioica* extracts have been investigated as potential anti-infective agents, also capable of ameliorating allergy symptoms and lowering skin irritability [27, 28]. Another field of primary relevance in which this plant finds application is that of women’s health, as it may help in counteracting menstruation symptoms, and it has also been reported to lessen the severity of hormonal shift through menopause. These effects are likely due to the coagulant effect and to the presence of hormone-like molecules [10].

With this review, we aim at providing an updated overview on the role of *U. dioica* extracts and components in counteracting neurodegeneration. In fact, mounting evidence shows that *U. dioica* can improve memory function and compounds isolated from this plant, such as the flavonoid rutin, improve cognition and reduce chronic stress-related dysfunctions of the central nervous system (CNS) in animal models [29, 30]. Nevertheless, this field has been only explored rather recently, but it would represent an attractive option from the point of view of medicinal chemists.

For the preparation of this review, the most relevant recent studies on neuroprotective effects of *U. dioica* were retrieved by using PubMed (pubmed.ncbi.nlm.nih.gov) and Scopus (scopus.com) databases. The literature-based screening was conducted using keywords such as *Urtica dioica* AND neuroprotective effects, *Urtica dioica* AND neurodegenerative disorders, *Urtica dioica* AND neurodegeneration, *Urtica dioica* AND pre-clinical trials, *Urtica dioica* AND clinical trials. More than 80 scientific papers were considered following this literature search.

## The neuroprotective potential of *U. dioica*

One of the major concerns for human health in modern societies is related to the increasing incidence of neurodegenerative diseases. AD, PD, HD, and other degenerative diseases are generally characterized by a loss of synapsis functioning and of neuromuscular connections. These events lead, to different extents, to memory issues and to a broad systemic neurological malfunction [31, 32].

It is now generally accepted that inflammation and oxidative stress play a primary role in neurodegenerative diseases [33–36]. In this connection, *U. dioica* and its extracts are being investigated as potential neuroprotective agents to combat neurodegeneration in light of its composition in terms of bioactive ingredients that act as anti-inflammatory and antioxidant agents [24, 37–39]. In particular, Esposito et al. recently reviewed the phytochemical composition of *U. dioica* in terms of bioactive components, thus the reader is invited to refer to this contribution for more details on this topic [14]. In this connection, *U. dioica* contains boron, an element which is thought to influence the levels of estrogen, a hormone that has a role in inflammation and short-term memory functioning [40]. From the point of view of the involved molecular mechanisms, the anti-inflammatory properties of *U. dioica* leaves extract can be mainly explained by the activation of NF-κB [41] and decreased generation of prostaglandin D2 (PGD2). Additionally, extracts from this plant showed a notable synergistic effect when used in combination with nonsteroidal anti-inflammatory drugs, leading to a reduction in C-reactive protein (CRP) levels [42].

As anticipated, *U. dioica* and its extracts were widely investigated for several potential therapeutic applications. In particular, Esposito et al. reviewed the reports on the anticancer activity of *U. dioica*-derived natural products [14], while Kregiel et al. presented a wider overview on the antimicrobial, analgesic, anti-inflammatory properties of *Urtica* species [40]. On the other hand, although Jaiswal and Lee reviewed the antioxidant properties of *U. dioica*, also considering the effects on brain [24], no comprehensive overview was reported to date concerning the state of the art of *U. dioica*-based remedies in the field of neuroprotection. In this connection, the current review is focused on the reports related to the potential role of *U. dioica* against neurodegenerative diseases.

### Computational studies

To date, only few studies based on the use of computational tools for the in silico investigation of compounds extracted from *U. dioica* were reported in the literature. Additionally, most of them are not directly related to the field of neurodegeneration but were carried out to support its more traditional role as an anti-infective agent. For example, in the past decade, Rao et al. investigated the binding of components from *U. dioica* towards FtsZ, a protein involved in bacterial cell division, using AutoDock Vina. The authors highlighted (-)-pinoresinol, isorhamnetin-3-*O*-neohesperidoside, lutein, olivil and rutin as the most promising compounds based on docking score [43]. More recently, Upreti et al. studied the interaction of bioactive molecules from *U. dioica* with angiotensin converting enzyme 2 (ACE2) in the context of the search for remedies against SARS-CoV-2. The authors, using PyRx virtual screening tool based on AutoDock 4.2 and Vina, proposed beta-sitosterol as the most promising candidate of the set [44]. Similarly, agglutinin from *U. dioica* was also proposed as a potential agent combating the interaction of the spike protein with ACE2 as demonstrated by protein–protein docking, molecular dynamics and in vitro studies [28].

**Fig. 1:**
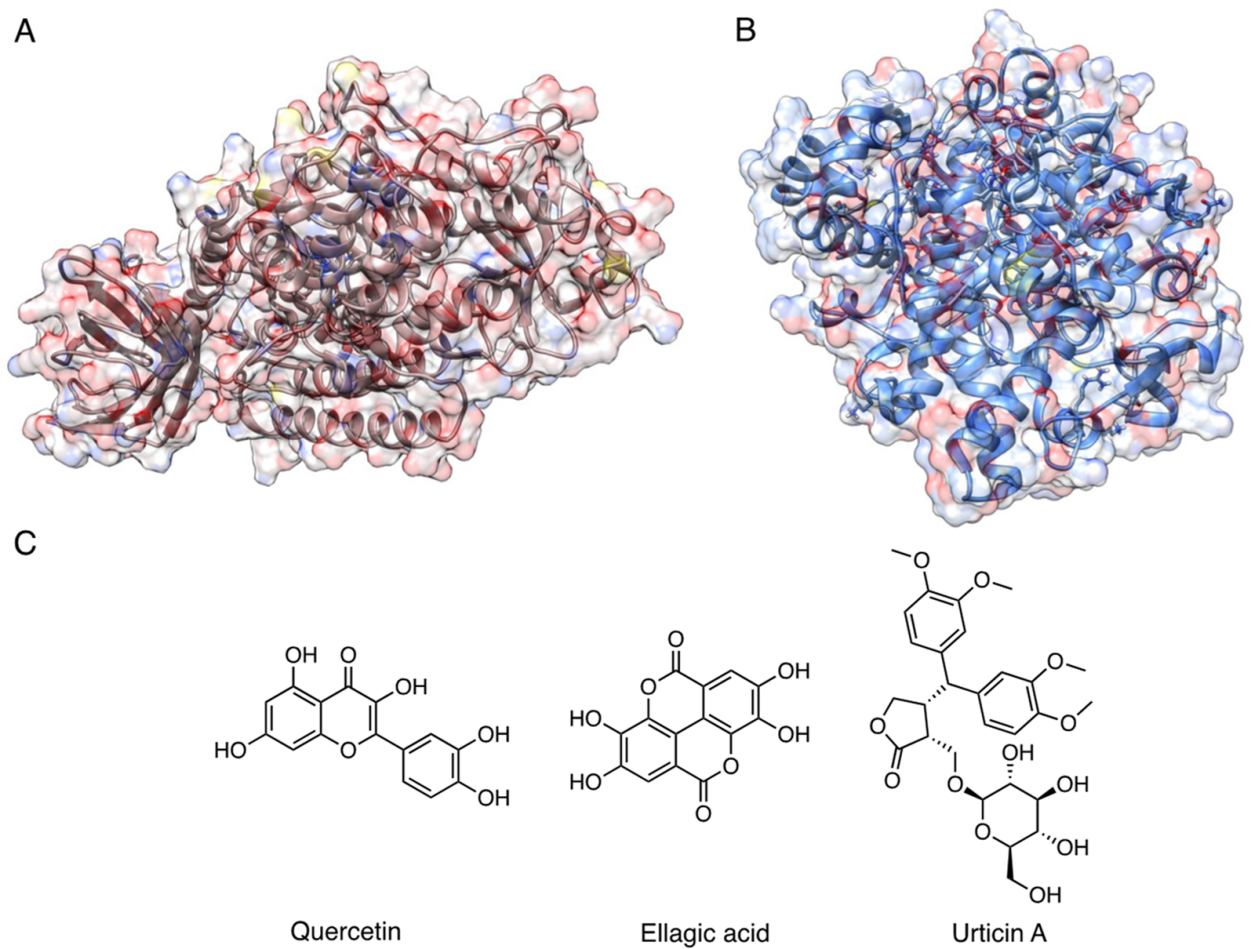
3D structures of 5-LOX (**A** PDB ID: 3O8Y) and of COX-2 (**B** PDB ID: 5KIR) [42]; (**C**) chemical structures of the compounds from *U. dioica* highlighted as potential anti-inflammatory agents according to computational studies

On the other hand, the study of Vyshnevska et al. better relates to the focus of this review, as it is more directly oriented towards the identification of anti-inflammatory agents in *U. dioica*. The authors considered macromolecular targets involved in chronic inflammation, and pro-inflammatory enzymes 5-lipoxygenase (5-LOX) and cyclooxygenase-2 (COX-2) in particular (Fig. 1A, B). In their study they included the bioactive molecules from *U. dioica* and other plants known to posses anti-inflammatory activity, such as *Foeniculum vulgare*, *Acorus calamus*, *Potentilla palustris*, *Petroselinum crispum*, *Inula helenium*, *Bergenia crassifolia*, *Veronica officinalis*, *Carum carvi*, *Levisticum officinale*, *Cichorium intybus*, *Capsella bursa-pastoris*, and *Equisetum arvense*. Among the studied compounds and following the docking carried out using AutoDock Vina, the authors identified some polyphenols as promising binders [45]. This aspect was also described by Idris et al. in their contribution in which they investigated polyphenols as ligands for 5-LOX and COX-2 [46]. For example, quercetin, which is present in *U. dioica* also in its glycosylated forms, was highlighted as a potential interactor of 5-LOX and COX-2 with anti-inflammatory activity [47]. Ellagic acid represent another example of polyphenolic compound contained in *U. dioica* [14]. The molecule was highlighted as a potential hit by this screening [45]. Similarly, a compound named urticin A, a secolignal isolated from *Urtica* rhizomes [48], was described among the best potential binders for both 5-LOX and COX-2, according to computational studies [45]. The structures of the cited compounds are depicted in Fig. 1C.

As it can be easily foreseen, for a compound to have a pharmacological effect in the CNS, it has to cross the blood–brain barrier (BBB). This is a limiting aspect for several natural compounds, but computational studies can aid the screening for CNS-targeting drug-like compounds. In fact, lipophilicity and permeation can be calculated in the context of ligand-based computational studies, in order to exclude false positives form subsequent development steps [49]. In this connection, the potential of quercetin in exploiting a therapeutic role within CNS has been recently discussed. Its pharmacological application in CNS diseases has been highlighted, and it is also enhanced by advanced formulation and delivery technologies, such as nanoformulations, which can boost BBB permeation [50]. Similarly, also the effects of ellagic acid in CNS have been reviewed [51].

### Preclinical studies

**Fig. 2:**
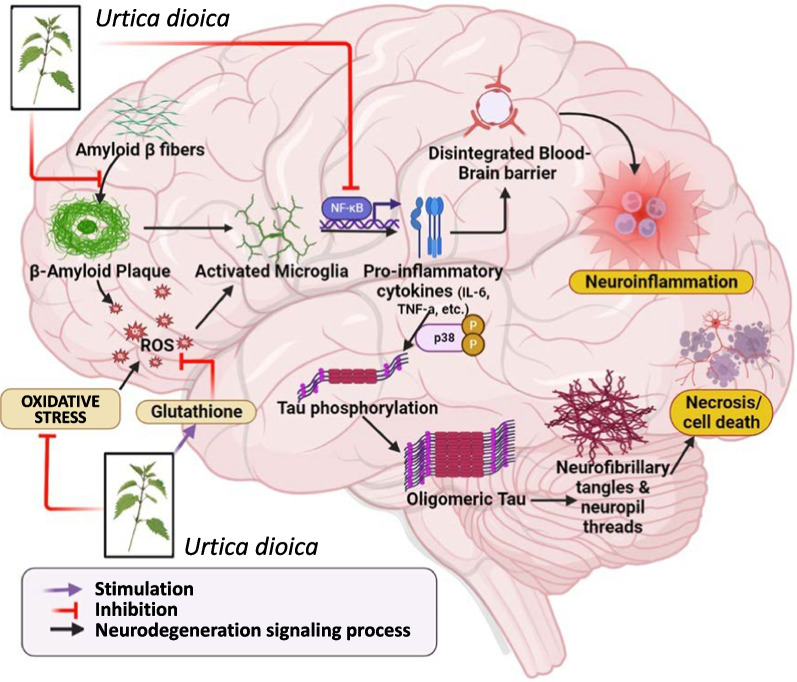
Potential neuroprotective mechanism of action of *U. dioica* and of its components

Neurodegenerative diseases are nowadays becoming a rising challenge for human health. AD, PD and HD are the most common examples, but other disorders have been described, that are characterized by impairment of memory, degeneration of neuromuscular connections and a systemic neurological impairment, although with different symptoms [52, 53]. Several medicinal plants and their bioactive components have been proposed as remedies or as adjuvant treatments to combat neurodegeneration [54–60]. In this context, recent reports support the potential of *U. dioica* and its extracts against neurodegenerative diseases in several different models [61]. A graphical representation resuming the pathways involved the potential neuroprotective effects of *U. dioica* is shown in Fig. 2, and the different molecular mechanisms that have been investigated and the experimental models will be explored in this section of the review.

*Urtica dioica* and its extracts have been investigated in vitro for the anti-inflammatory and antioxidant potential of the bioactive components, which are mainly related to the rich variety of polyphenolic compounds present in this plant [23, 41].

Concerning contributions reporting the preclinical effects of *U. dioica* in combating neurodegeneration in vivo, the extract rich in antioxidant species showed neuroprotective effects in a 1-methyl-4-phenyl-1,2,3,6-tetrahydropyridine (MPTP)-induced PD model [62]. Bisht et al. reported that the administration of *U. dioica* (80, 40, and 20 mg/kg) for 2 weeks enhanced motor coordination, behavioral performance, and decreased concentration of prooxidant species. Moreover, *U. dioica* (80 mg/kg) enhanced the neuroprotective action of minocycline [63]. Additionally, the plant reduced pro-inflammatory proteins such as interleukin-1β (IL-11β) and tumor necrosis factor-α (TNF-α). On the other hand, it improved the levels of catalase (CAT). Overall, the author noted that *U. dioica* shows a remarkable property in the regulation of the antioxidant system and attenuates the impairments induced by inflammatory markers [62].

It has also been reported that diets containing *U. dioica* ameliorate the conditions of Wistar rats with *N*-methyl d-aspartate (NMDA)-induced inflammation, an event which affects the function of the brain and induces neurodegenerative disorders. NMDA also impairs memory abilities, but *U. dioica* induced protective effects in this context. Eventually, as shown by EPR measurements, *U. dioica* supplementation markedly reduced free radical accumulation from the cerebellum [29, 31, 64].

In the context of AD, there are genetic factors which are genes associated with a family history of the disease, and this relates to cases of familial AD, and sporadic AD [65]. An herbal extract consisting of *U. dioica* and two other medicinal plants (*Tanacetum vulgare* and *Rosa canina*) markedly improved the expressions of genes that play a crucial role in the pathogenesis of sporadic AD. More specifically, the administration of the herbal extract containing *U. dioica* (20 mg/kg) enhanced spatial learning and memory in a rat model of sporadic AD [39].

**Fig. 3:**
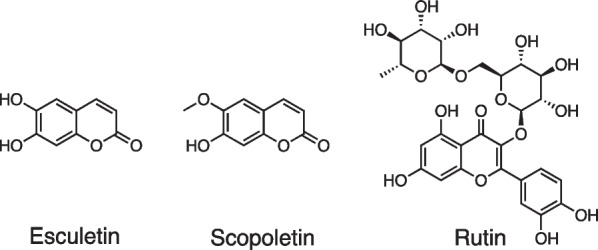
Chemical structures of the compounds contained in *U. dioica* that showed anti-inflammatory and antioxidant potential in diabetes mice model

Leaf extract of *U. dioica* containing high concentration of quercetin (Fig. 1C), esculetin, scopoletin and rutin (Fig. 3) exerted anti-inflammatory and antioxidant properties in the hippocampus of mice with STZ-induced diabetes [9, 66]. Additionally, the quantity of astrocytes in the hippocampus of diabetic rats decreased following administration of *U. dioica* extracts [67].

Qayyum et al. reported the antihypertensive effects of *U. dioica* and of isolated fractions of the extract. These showed an antihypertensive effect which is related to the induced vasodilation. The mechanisms behind this event include the release of nitric oxide (NO) and the interference with calcium homeostasis in the blood vessels. The authors applied these findings to support the use of *U. dioica* as a potential treatment for hypertension [9], but the positive effects on microvasculature also represent an attractive strategy to ameliorate neurodegeneration conditions [68].

Diabetic animal models are often used to study neurodegenerative progression. In this context, another study involving the leaf extract of *U. dioica* showed that the plant exhibited neuroprotective effects by regulating various cascades, which are resumed in Fig. 2. *U. dioica* reduces the mRNA levels of iNOS, whereas it increases the mRNA levels of TrKB, Bcl2, BDNF, and cyclin D1. As anticipated, it also decreases the expression of TNF-α in various hippocampal regions. Furthermore, it ameliorates neuronal injury, and an overall decrease of DNA fragmentation has been observed in the hippocampus of diabetic rats. In their study, Patel et al. concluded that the extracts obtained from leaves of *U. dioica* may be effective in reducing anxiety and depression-like conditions related to diabetes [69]. In this context, the *U. dioica* extract was reported to reduce loss of granule cell in the dentate gyrus and hippocampus cells in diabetic rats [70]. *U. dioica* extract also reversed memory impairments in different mouse models of dexamethasone-induced diabetes [66, 67, 69–71]. More specifically, the already mentioned flavonoid glycoside rutin was reported to exert neuroprotective action in the retina of diabetic rats due to its antioxidant potential [72].

In another study focused on diabetes and cognitive impairment, *U. dioica* administered for 6 weeks to rats with STZ-induced diabetes (50 mg/kg) in combination with exercise attenuated insulin signaling impairments, neuroinflammation, cognitive impairments, hippocampal oxidative stress, and apoptosis, further supporting the potential of the plant in this context [73].

Stimulation of NF-κB, a protein that mediates oxidative injury through stimulation of pro-inflammatory markers, was shown to be inactivated through abrogation of its nuclear translocation by both *U. dioica* supplementation and exercise [29]. In particular, a hydroalcoholic *U. dioica* extract showed neuroprotective action in a scopolamine-induced memory impairment model (15 mg/kg). According to behavioral studies, administration of *U. dioica* (20, 50, and 100 mg/kg) improved cognitive function in the rodent model [74].

Additionally, the neuroprotective role of *U. dioica* on STZ-induced model of diabetes in rats with impaired hippocampus was investigated. Treatment with the hydroalcoholic extract of *U. dioica* (45 and 50 mg/kg) improved microglial density and lead to a thickening of the hippocampal pyramidal layer. In the same model, treatment with *U. dioica* induced an increase in the levels of growth associated protein (GAP)-43, enhanced hippocampus plasticity, reduced the levels of adenylyl cyclase-associated protein-1 (CAP-1) and enhanced memory and learning functions. Thus, the study highlighted that *U. dioica* may contribute in reducing the central neural complications of diabetes [75].

According to another report, *U. dioica* improved brain functions in a propionic acid-induced autism rat model. Oral administration of *U. dioica* root extract (50 mg/kg) ameliorated behavioral deficits, along with enhancement of metabolism and functioning of monoaminergic system [76].

In a recent comparative study between *U. dioica* and two other plants, namely *Viola spathulata* and *Lamium album*, the effects in terms of protective properties on endoplasmic reticulum stress in rat were investigated [77]. The plant extracts remarkably reduced target gene splicing in focal cerebral ischemic animals. More specifically, this effect observed for *U. dioica* was linked to the presence of antioxidants in the plant. In the same study, *U. dioica* ameliorated defective brain inflammatory mechanisms and stroke-induced cellular stress [77].

### Clinical studies

In a very recent report, Khosravi et al. showed that *U. dioica* root extract can effectively treat tinnitus symptoms. More specifically, a double-blind clinical trial involving 103 individuals (53 cases and a control group of 50 subjects) was considered and the subjects received 100 mg of Neurotec (a combination of *Rosa canina*, *U. dioica* and *Tanacetum vulgare*) daily for 3 months. Significant improvements in terms of the symptoms such as tinnitus loudness, severity, tinnitus perception, sleep alteration, mood disturbance and overall quality of life were recorded, suggesting that *U. dioica* could improve auditory nerve function [78].

In another clinical trial, 99 individuals who had experienced their first acute ischemic stroke (50 in the control group and 49 participants to the treated group) were considered. The IMOD™ group, received setarud (IMOD™), a mixture of selenium-enriched extracts from the plant species *T. vulgare*, *R. canina*, and *U. dioica*. In detail, this phytotherapeutic agent contains plant-based ingredients which are characterized by anti-inflammatory and immunoregulatory activities, and selenium, that is a known antioxidant. Extracts from *U. dioica* reduced myeloid dendritic cell maturation and T cells response. Moreover, it decreased TNF-α, interferon and IL-2 levels, as demonstrated by several in vitro and in vivo experiments performed on both human and animal models. The results showed that *U. dioica* improved the profile of main inflammatory markers; therefore, it might be considered as a therapeutic option in ischemic strokes due to its neuroprotective effects [79].

In a randomized placebo-controlled trial, a formulation called Rosaxan or MA212 consisting of *U. dioica* leaf extract (160 mg), fruit puree of *R. canina* L. (20 g), root extract of *Harpagophytum procumbens* (108 mg), and fruit juice concentrate of *R. canina* (20 g) was administered to patients. Improvements of Western Ontario and McMaster Universities Arthritis Index, which include mental and physical quality of life, were observed [31, 80].

## The perspective of the medicinal chemist

*Urtica dioica* is a widely studied plant and several contributions focusing on the potential applications of its extracts or components in medicinal chemistry appeared in the literature throughout the years.

For a comprehensive overview of the chemical profile of bioactive constituents of *U. dioica* and of the reported pharmacological applications, the reader is invited to refer to the recent review article by Taheri et al. [26]. The authors systematically reported the main bioactive class of compounds comprehending lignans, sterols, fatty acids, phenols, flavonoids, alkaloids and terpenoids. Contemporarily, the authors efficiently resumed in vitro, preclinical and clinical evidence supporting the potential of *U. dioica* extracts and components as anticancer, antibacterial, antiviral, anti-inflammatory, antioxidant and antiaging agents acting through a combination of mechanisms [26]. Similarly, Khan et al. reported the phytochemical characterization of essential oils of *U. dioica* with potential antioxidant, phytotoxic and antibacterial activities, that were mainly attributed to lipophilic compounds detected in the extracts and studied by means of computational and in vitro techniques [81].

On the other hand, even if *U. dioica* is commonly used in traditional medicine and several applications have been reported and reviewed previously [82, 83], its potential in the field of neurodegenerative disease has not been fully explored yet, and only few reports systematically overviewing the neuroprotective action of *U dioica* extracts and constituents can be retrieved [13].

**Fig. 4:**
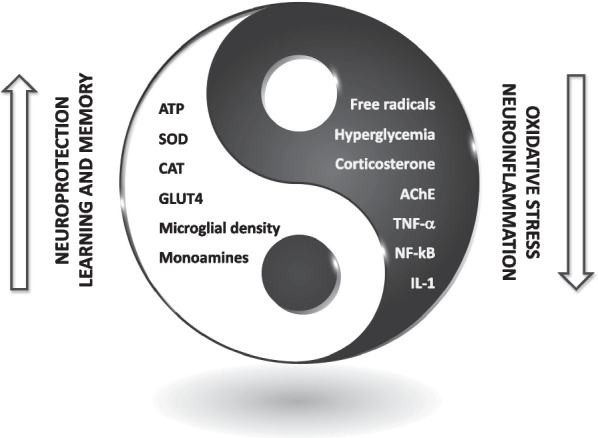
Neuroprotective potential of *U. dioica* and proposed molecular mechanisms: an equilibrium between different events leading to neuroprotection

The neuroprotective effect of *U. dioica* can be connected with a plethora of different molecular mechanisms, but the role of its components against inflammation and oxidative stress plays a fundamental role. A simplified overview of the involved processes, overviewed in this contribution, is reported in Fig. 4.

The anti-inflammatory potential of *U. dioica* is associated with its inhibitory action on enzymes involved in inflammation, such as COXs. Additionally, the contribution of the chronic inflammation hallmark NF-κB must be taken into account. NF-κB leads to increased production of pro-inflammatory genes and its activity is triggered by a wide range of inflammatory responses, such as infections caused by bacteria and viruses and other stressors. In this context, NF-κB regulates the expression of many genes, including those that produce inflammatory cytokines [84]. The anti-inflammatory properties of *U. dioica*, and of its leaf extracts in particular, are partly due to its ability to inhibit NF-κB activation. In particular, *U. dioica* components have an inhibitory action on NF-κB by preventing of degradation of its inhibitor [41]. Additionally, it has been demonstrated that treatment with aqueous *U. dioica* extracts can block the lipopolysaccharide-induced production of nitric oxide in macrophages. And decrease the release of pro-inflammatory substances such as cytokines [85]. Moreover, the anti-inflammatory and immunostimulant potential may be mediated by the interference with Toll-Like Receptor 4 (TLR4) pathway [86].

Thus, provided that it has been proved that *U. dioica* constituents can cross the BBB and exert their activity within the CNS, the multi-target character of this plant must be stressed. In fact, while COXs, NF-κB and other mediators represent macromolecular targets that have been widely studied in the field of drug discovery, the strength of the *U. dioica* extract approach lies on the synergistic action of its phytocomplex.

An overview of the proposed molecular mechanisms of neuroprotective activity of *U. dioica* is resumed in Table 1.

**Table 1:** Neuroprotective activity of *U. dioica* based on in vitro and in vivo studies

Plant parts	Extract or preparation	Model/cell lines	Mechanism	References
Leaves	Hydroalcoholic extract, 1 mg/mL	Caco-2 cell lines	↓Maltase, ↓sucrase, ↓lactase	[23]
Root and leaves (capsules)	200–500 mg/capsule	Swiss Albino rats	Normalized Hsp70 and Tau expression in cortical and hippocampal compartments to protect the brain from SCOP-induced memory deficits, Restored ATP level, ↓AMP, ↓ADP	[61]
Aerial parts	Ethanolic extract, 100 mg/kg	Male Wistar rats	↓ER stress, ↓XBP-1 gene splicing	[77]
Root and leaves	1–250 mg/kg	Swiss Albino rats	↑Monoaminergic system, ↑bioenergetics	[76]
Aerial parts	Hydroalcoholic extract, 50 mg/kg	Male Wistar rats	↑Neural-microglial density, ↑GAP-43 protein level, ↓CAP-1 protein level, ↑learning and memory	[75]
Aerial parts	Hydroalcoholic extract, 20–100 mg/kg	Male Wistar rats	↓AChE, ↓MDA conc, ↑thiol conc., ↑SOD activity, ↑CAT activity	[74]
Leaves	Hydroalcoholic extract, 20–80 mg/kg	Male Wistar rats	↑TNF-α, ↑IL-β, restored the level of dopamine, ↓mito-oxidative damage, ↓neuroinflammation	[62]
Rhizomes	Methanolic extract, and fractions,	Rabbits and Sprague–Dawley rats	Blocking of Ca^2+^ channel activity, NO-mediated vasorelaxation effects	[9]
Leaves	Ethanolic extract, 20 mg/kg/day	Male Wistar rats	↓PSEN1, ↑Learning and memory	[39]
Leaves	Hydro-alcohol extract, 50 mg/kg	Swiss Albino mice	↓Cholinergic dysfunction, ↓oxidative stress	[69]
Leaves	Hydroalcoholic extract, 50–100 mg/kg	Swiss Albino mice	↓Hyperglycemia, ↓plasma corticosterone, ↓oxidative stress, ↑memory, ↑GLUT4 mRNA expression	[71]
Leaves (in combination)	250–375 mg/day	Human trials (58 male and 41 females)	↓TNF-α, ↓IL level, ↑hsCRP level, ↑IL-6	[79]
Leaves	1% w/w for 8 weeks	Male Wistar rats	↓NF-kB activation in NMDA lesion, ↓Free radical concentration, ↑DNA binding of AP-1	[64]
Leaves	Hydroalcoholic extract, 375 µg	HeLa, L929 cell lines	↓NF-kB activation, ↓cytokine production	[41]

*HSP* heat shock protein, *SCOP* scopolamine, *AMP* adenosine monophosphate, *ADP* adenosine diphosphate, *ER* endoplasmic reticulum, *XBP-*1 X-box binding protein 1, *GAP-43* growth associated protein, *CAP-1* cyclase associated protein, *AChE* acetylcholinesterase, *MDA* malondialdehyde, *SOD* superoxide dismutase, *CAT* catalase, *TNF-α* tumour necrosis factor-alpha, *IL-1β* interleukin-1 beta, *NO* nitric oxide, *PSEN-1* presenilin-1, *GLUT-4* glucose transporter type-4, *hsCRP* high-sensitivity C-reactive protein, *NF-kB* nuclear factor-kB, *NMDA N*-methyl-d-aspartic acid

## Conclusions

*Urtica dioica* has been adopted and used both as a source of nutrients and as a traditional medicine for decades. It is abundant in phytonutrients and contains a variety of bioactive phytoconstituents, including polyphenols and flavonoids.

Neuroprotective efficacy of medicinal plants can be achieved by exhibiting various mechanisms such as antioxidant activity, inhibition of inflammation and preventing accumulation of polyubiquitinated protein aggregates in brain and enhancing protective signaling. It has been demonstrated that *U. dioica* has provided neuroprotective activity via modulation of different inflammatory and biochemical markers (TNF-α, IL-1β, NF-kB, GSH, CAT, etc.) and highlights the significant potential in the management of neuroinflammation.

According to numerous computational, in vitro*, *in vivo, and clinical investigations, *U. dioica* represents a promising herb with neuroprotective potential, especially for neurodegenerative disorders associated with diabetes and AD. However, further research is required to completely comprehend the molecular mechanism and signaling pathway underlying the anti-inflammatory and antioxidant effects of *U. dioica*. In conclusion, the identification of targets related to the signaling pathway influenced by *U. dioica* could pave the way for a future drug development for treatment of neurodegenerative disorders.

## Data Availability

No new data was generated for this review.
